# Geriatric Interprofessional Education Theoretical Framework

**DOI:** 10.1093/geroni/igab046.001

**Published:** 2021-12-17

**Authors:** Jennifer Drost

**Affiliations:** Summa Health System, Akron, Ohio, United States

## Abstract

The literature is lacking in theoretically grounded techniques to teach interprofessional skills specific to caring for older adults. This presentation details how Wagner’s Chronic Care Model and the Constructivist/Active Learning theoretical frameworks were used in the design of an interprofessional education. The content of the education was modeled after Wagner’s chronic illness care model that advocates changes in processes and organizational structures to promote interprofessional team practice. The educational intervention follows a Constructivist/Active learning framework delivered in a simulation format. Constructivist approaches encompass active learning and guided experiential learning procedures, methods well-suited to our scaffolded simulation educational experience.

